# An Improved UWB/IMU Tightly Coupled Positioning Algorithm Study

**DOI:** 10.3390/s23135918

**Published:** 2023-06-26

**Authors:** Airu Zou, Wenwu Hu, Yahui Luo, Ping Jiang

**Affiliations:** College of Mechanical and Electrical Engineering, Hunan Agricultural University, Changsha 410128, China; zar@stu.hunau.edu.cn (A.Z.); luoyh@hunau.edu.cn (Y.L.);

**Keywords:** ultra-wide band, tightly coupled positioning, non-line-of-sight, extended Kalman filter

## Abstract

The combination of ultra-wide band (UWB) and inertial measurement unit (IMU) positioning is subject to random errors and non-line-of-sight errors, and in this paper, an improved positioning strategy is proposed to address this problem. The Kalman filter (KF) is used to pre-process the original UWB measurements, suppressing the effect of range mutation values of UWB on combined positioning, and the extended Kalman filter (EKF) is used to fuse the UWB measurements with the IMU measurements, with the difference between the two measurements used as the measurement information. The non-line-of-sight (NLOS) measurement information is also used. The optimal estimate is obtained by adjusting the system measurement noise covariance matrix in real time, according to the judgment result, and suppressing the interference of non-line-of-sight factors. The optimal estimate of the current state is fed back to the UWB range value in the next state, and the range value is dynamically adjusted after one-dimensional filtering pre-processing. Compared with conventional tightly coupled positioning, the positioning accuracy of the method in this paper is improved by 46.15% in the field experimental positioning results.

## 1. Introduction

With the development of science and technology, the application of navigation and positioning technology is being increasingly researched [1,2,3], and the methods of navigation and positioning are being gradually diversified. At present, common positioning technologies include global positioning system (GPS) [4,5,6,7], BeiDou navigation satellite system (BNSS) [8,9], inertial navigation system (INS) [10,11,12], UWB (ultra-wide band, UWB) [13,14,15,16,17], machine vision navigation systems [18,19,20,21,22] and LIDAR [23,24,25,26]. Among them, ultra-wide band technology stands out among many positioning technologies due to its low power consumption, strong anti-interference, high penetration power and high positioning accuracy, and its application is becoming more and more widespread. Zhao Ziyu et al. [27] developed a UWB positioning photogrammetry tree meter with a real-time positioning function and forest stand photogrammetry to achieve efficient and accurate determination of forest sample sites. Lin Xiangze et al. [28] developed an indoor positioning test platform of UWB technology applicable to a greenhouse environment to resolve the problem of low positioning accuracy of agricultural vehicles under a greenhouse environment. However, the experimental platform does not consider the impact of non-line-of-sight factors on UWB positioning, and does not consider the lack of positioning stability of a single positioning method.

Non-line-of-sight (NLOS) refers to the prolongation of signal reception time due to signal occlusion by obstacles, which in turn degrades the positioning accuracy of the system. Sheng Kunpeng et al. [29] proposed a modelling method that takes into account the angle of incidence of the UWB pulse signal to address the problem of localization bias in the non-visual range environment of ultra-wideband systems. Kong et al. [30] proposed a bi-directional search algorithm based on maximum correlation, minimum redundancy and minimum computational cost for the identification of NLOS states, which can effectively identify NLOS and LOS states by setting constraint thresholds for maximum evaluation metrics and computational cost, as well as the best channel impulse response (CIR) feature set for the blocking category. Ansaripour et al. [31] proposed a real-time attitude estimation system called ViPER+ in order to eliminate the effect of NLOS signals, which overcomes the NLOS situation and accurately determines the boundaries of heavy construction equipment by multiple UWB tags attached to the equipment surface, introducing prior to localization an input correction method to correct the inputs to the localization algorithm.

At once, single-mode positioning and navigation methods work unstably due to their own shortcomings, so two or more positioning methods can be selected for combined positioning. Ultra-wide band positioning is highly accurate, with low power consumption and high penetration, unaffected by weak differential signal environment and commonly used in complex environment positioning [32], but its positioning accuracy is susceptible to random errors and NLOS (non-line-of-sight) factors [33,34]; the inertial measurement unit (IMU) solves the target velocity and positioning through gyroscopes and accelerometers and is not interfered with by external factors, and its positioning is autonomous, but due to its positioning nature, it is not suitable for long-duration positioning due to the accumulated errors. Therefore, the two positioning methods of UWB and IMU are tightly coupled [35,36], and can play the role of mutual constraints and complement each other.

In summary, this paper proposes an improved method for tightly coupled UWB/IMU localization based on EKF (Extended Kalman Filter, EKF) using the localization characteristics of both UWB and IMU, with UWB as the research object and IMU as the auxiliary object. The Kalman filter is used to pre-process the original UWB range values via one-dimensional filtering, and the EKF is used to fuse the UWB measurement data with the IMU measurement data to obtain tightly coupled positioning. At the same time, the current UWB range values are judged to have NLOS errors according to the tightly coupled positioning results, and the final positioning is solved by dynamically adjusting the measurement noise covariance matrix according to the judgment results. The simulation environment and position estimation experiments in the real environment are designed to verify the algorithm’s effectiveness.

## 2. Construction of a Positioning Measurement System and Acquisition of Measurement Information

### 2.1. Introduction to UWB Ranging Principle

The UWB ranging principle used in this experiment is based on the bilateral ranging principle TW-TOF (Tow-way ranging time of flight, TW-TOF), which is a two-way ranging technique that uses the time of flight of a data signal round trip between a pair of transceivers to measure the distance between two points.

**Figure 1:**
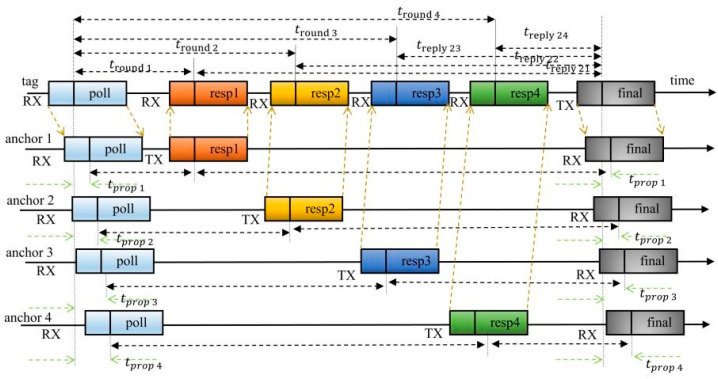
Schematic diagram of UWB bilateral ranging principle.

The UWB tag sends a poll message to the four UWB anchors, the four UWB anchors receive the poll message and reply with a response message back to the UWB tag, and the tag receives the response message and then sends a final message as a ranging cycle for ranging. The advantage of this is that it reduces the number of messages sent by the UWB tag and reduces the power consumption of the tag. The interaction between the tag and the UWB anchors and the ranging schematic are shown in Figure 1. In Figure 1, RX means “receiver” and TX means “transmitter”.

By using the principle of Figure 1, the time from the four UWB anchors to the tag is obtained, and the resulting time is shown in Equation (1):

(1) $$\{\begin{array}{c} t_{\mathrm{prop}1}=\frac{t_{\mathrm{round}11}\times t_{\mathrm{round}21}-t_{\mathrm{reply}11}\times t_{\mathrm{reply}21}}{t_{\mathrm{round}11}+t_{\mathrm{round}21}+t_{\mathrm{reply}11}+t_{\mathrm{reply}21}}\\ t_{\mathrm{prop}2}=\frac{t_{\mathrm{round}12}\times t_{\mathrm{round}22}-t_{\mathrm{reply}\;12}\times t_{\mathrm{reply}22}}{t_{\mathrm{round}12}+t_{\mathrm{round}22}+t_{\mathrm{reply}12}+t_{\mathrm{reply}22}}\\ t_{\mathrm{prop}3}=\frac{t_{\mathrm{round}13}\times t_{\mathrm{round}23}-t_{\mathrm{reply}13}\times t_{\mathrm{reply}23}}{t_{\mathrm{round}13}+t_{\mathrm{round}23}+t_{\mathrm{reply}13}+t_{\mathrm{reply}23}}\\ t_{\mathrm{prop}4}=\frac{t_{\mathrm{round}14}\times t_{\mathrm{round}24}-t_{\mathrm{reply}14}\times t_{\mathrm{reply}24}}{t_{\mathrm{round}14}+t_{\mathrm{round}24}+t_{\mathrm{reply}14}+t_{\mathrm{reply}24}} \end{array}$$

In Equation (1), $t_{\mathrm{prop}j}$ is the time from the UWB tag to the UWB anchors; $t_{\mathrm{round}1j}$ is the return time; and $t_{\mathrm{reply}1j}$ is the response time, where 1, 2, 3 and 4 represent four UWB anchors.

According to Equation (2), multiplying the time of the four UWB anchors by the speed of the electromagnetic wave gives the ranging information of the four UWB signals, where *d* is the measured distance between the UWB tag and the UWB anchor and *c* is the electromagnetic wave velocity.

(2) $$d=t_{\mathrm{prop}j}\cdot c$$

### 2.2. Acquisition of Measurement Information

**Figure 2:**
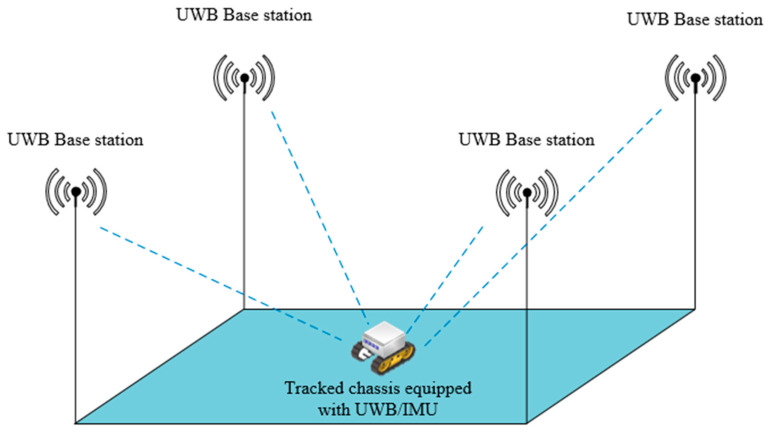
Schematic diagram of the UWB anchors construction for the UWB positioning system.

This section describes the positioning system build and the way the raw data was acquired in the field experimental study. UWB positioning starts with a systematic UWB anchors build for the activity range of the target node, as shown in Figure 2.

**Figure 3:**
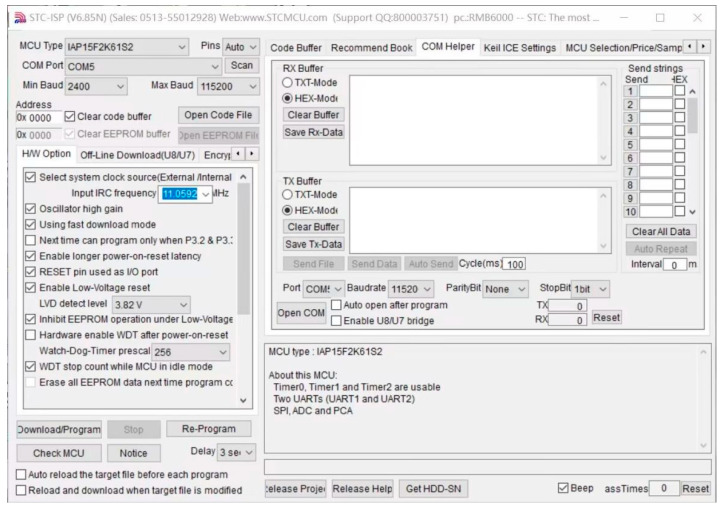
Raw measurement data acquisition interface.

This paper uses STC-ISP software to obtain the positioning sensor data. STC-ISP is a microcontroller download and programming software, designed for STC series microcontrollers, which can download STC89 series, 12C2052 series and 12C5410 series STC microcontrollers and is easy to use. Figure 3 shows the interface for acquiring the original measurement data of the positioning sensor. The distance information measured by the UWB signal and the acceleration information and angular velocity information measured by the IMU are obtained in the RX buffer in the figure. Before acquiring the data, the baud rate is set to 11,520, the microcontroller model is selected, power is applied to the positioning module and the Bluetooth module is connected to the PC via the USB serial port so that the sensor measurement data can be acquired in real time.

In this paper, the collected raw data is processed through a Python program for serial data to change the measurement data format, and the measurement data is subsequently analyzed in the Matlab platform to obtain the positioning data of the target node.

## 3. Positioning Principle

### 3.1. Kalman Filtering Pre-Processed UWB Range Values

In this paper, the Kalman filter is used to pre-process the range values and suppress random system errors and some non-line-of-sight range errors that cause abrupt changes in UWB range values. Use the difference $\Delta d_{i}\left(i=1,2,\cdots ,n\right)$ between the range values of moments t−1 and moments t as the measurement vector, set $x_{1}=0$ as the initial state and establish a discrete state model for the range value filtering.

(3) $$\left\{ \begin{array}{c} x_{t}=Ax_{t-1}+W_{t}\\ z_{t}=Hx_{t}+V_{t} \end{array}\right.$$

In Equation (3), A denotes the system state transfer matrix, $A=\left[\begin{array}{cc} 1 & dt\\ 0 & 1 \end{array}\right]$; H denotes the system measurement matrix and $H=\left[\begin{array}{cc} 1 & 0 \end{array}\right]$; $W_{t}$, $V_{t}$ are the process noise and measurement noise at the moment, respectively, and $W_{t}∼N(0,Q_{t}),V_{t}∼N(0,R_{t})$.

A priori estimation based on the discrete state model yields the predicted state $x_{t}^{-}$ and predicted covariance $P_{t}^{-}$

(4) $$x_{t}^{-}=Ax_{t-1}$$

(5) $$P_{t}^{-}=AP_{t-1}A^{T}+Q_{t}$$

where $x_{t-1}$ denotes the momentary t−1 optimal state estimate and $Q_{t}$ is the covariance matrix of the momentary t process noise. The Kalman gain $K_{t}$ is obtained from the system as:

(6) $$K_{t}=P_{t}^{-}H{\left(HP_{t}^{-}H^{T}+R_{t}\right)}^{-1}$$

The above equation shows that the prediction covariance $P_{t}^{-}$ Kalman gain $K_{t}$ is positively related, and the observation noise $R_{t}$ is inversely related to the Kalman gain $K_{t}$. Therefore, when the measurement vector error increases, the Kalman gain $K_{t}$ at the current moment will decrease. Updating state ${\hat{x}}_{t}$ and covariance $P_{t}$ based on the above expression yields the following:

(7) $${\hat{x}}_{t}={\hat{x}}_{t-1}+K_{t}\cdot (z_{t}-H(x_{t}^{-}-{\hat{x}}_{t-1}))$$

(8) $$P_{t}=(1-H\cdot K_{t})P_{t}^{-}$$

${\hat{x}}_{t}$ indicates the optimal estimate in the current state, and ${\hat{x}}_{t}$ and $P_{t}$ will participate in the a priori estimate at moment t+1 until the end of the filtering to obtain the UWB range value after the 1D filtering process. In this paper, the importance of one-dimensional filtering in tightly coupled positioning will be verified through simulated positioning comparison experiments.

### 3.2. Tightly Coupled Positioning Based on the NLOS Judgment Mechanism

In tightly coupled UWB/IMU positioning, the UWB suppresses the cumulative error of the IMU over time, while the IMU is not affected by the environment in a non-line-of-sight environment, which can mitigate the effect of the UWB from non-line-of-sight errors. In this paper, the difference between the measured values of UWB and IMU is used as the measurement information, while feedback correction is applied to the tightly coupled positioning system.

The IMU mainly consists of accelerometers and gyroscopes, which measure the acceleration and angular velocity in the carrier coordinate system and solve the position, velocity and altitude information of the target node by integration; the IMU model is shown in Equation (9)

(9) $$\left\{ \begin{array}{c} p_{k}^{N}=p_{k-1}^{N}+\Delta Tv_{k-1}^{N}+\frac{1}{2}\Delta T^{2}a_{k-1}^{N}\\ v_{k}^{N}=v_{k-1}^{N}+\Delta Ta_{k-1}^{N}\\ \psi_{k}=\psi_{k-1}+\Delta T\omega_{k-1}^{N} \end{array}\right.$$

where ΔT is the IMU sampling interval; $p_{k}^{N}$, $v_{k}^{N}$, and $a_{k}^{N}$ are the position, velocity, and horizontal acceleration under the N system at moment k; and $p_{k}^{N}={\left[p_{x}\;p_{y}\right]}^{T}$, $v_{k}^{N}={\left[v_{x}\;v_{y}\right]}^{T}$ and $\psi_{k}$ are the carrier attitude angles at moment k, respectively.

$a_{k}^{B}$ is the acceleration under the navigation and positioning system B, and needs to be converted to $R_{B}^{N}$ under the carrier positioning system N by means of a rotation matrix $R_{B}^{N}$; the rotation matrix $R_{B}^{N}$ is shown in Equation (10).

(10) $$R_{B}^{N}=\left[\begin{array}{cc} \cos\psi_{k} & -\sin\psi_{k}\\ \sin\psi_{k} & \cos\psi_{k} \end{array}\right]$$

This gives $a_{k}^{N}=R_{B}^{N}a_{k}^{B}$, and the tightly coupled state prediction equation is

(11) $$X(k)=FX(k-1)+BR_{B}^{N}\cdot U_{k}+w(k)$$

where $X(k)={\left[{\left(p_{k}^{N}\right)}^{T},{\left(v_{k}^{N}\right)}^{T},\psi_{k}\right]}^{T}$ is moment k’s state vector; $U_{k}=\left[\begin{array}{cc} a_{b}^{x} & a_{b}^{y} \end{array}\right]$ is moment k’s acceleration; w(k) represents process noise; and $w(k)\sim N(0,q_{k})$. F and B are:

(12) $$F=\left[\begin{array}{ccccc} 1 & 0 & \Delta T & 0 & 0\\ 0 & 1 & 0 & \Delta T & 0\\ 0 & 0 & 1 & 0 & 0\\ 0 & 0 & 0 & 1 & 0\\ 0 & 0 & 0 & 0 & 1 \end{array}\right]$$

(13) $$B=\left[\begin{array}{cc} {\left(\Delta T\right)}^{2}/2 & 0\\ 0 & {\left(\Delta T\right)}^{2}/2\\ \Delta T & 0\\ 0 & \Delta T\\ 0 & 0 \end{array}\right]$$

Moment k’s prediction covariance

(14) $$P_{k}^{-}=FP_{k-1}F^{T}+BR_{B}^{N}\cdot U_{k}\cdot {\left(BR_{B}^{N}\right)}^{T}+q_{k}$$

where during the initialization phase, the matrix *P*_0_ is expressed as:

(15) $$P_{0}=\left[\begin{array}{ccccc} 0.01 & 0.01 & 0.01 & 0.01 & \\ 0.01 & 0.01 & 0.01 & 0.01 & \\ 0.01 & 0.01 & 0.01 & 0.01 & \\ 0.01 & 0.01 & 0.01 & 0.01 & \end{array}\right]$$

Calculating Kalman gain $K_{k}$:

(16) $$K_{k}=P_{k}^{-}H{\left(HP_{k}^{-}H^{T}+r_{k}\right)}^{-1}$$

Here, $H_{k}$ denotes the system measurement equation

(17) $$H_{k}=\left[\begin{array}{ccccc} \frac{\partial d_{1,k}^{imu}}{\partial p_{x}^{N}} & \frac{\partial d_{1,k}^{imu}}{\partial p_{y}^{N}} & 0 & 0 & 0\\ \frac{\partial d_{2,k}^{imu}}{\partial p_{x}^{N}} & \frac{\partial d_{2,k}^{imu}}{\partial p_{y}^{N}} & 0 & 0 & 0\\ \ldots & \ldots & \ldots & \ldots & \ldots \\ \frac{\partial d_{i,k}^{imu}}{\partial p_{x}^{N}} & \frac{\partial d_{i,k}^{imu}}{\partial p_{y}^{N}} & 0 & 0 & 0 \end{array}\right]$$

where $d_{i,k}^{imu}$ denotes the distance from the positioning coordinates calculated by the IMU to the ith UWB anchor at moment k, and the tightly coupled measurement equation is expressed as

(18) $$Z(k)=H_{k}x(k)+v(k)$$

with observations $Z(k)={\left[\Delta d_{k,1}\;\Delta d_{k,2}\cdots \Delta d_{k,Num}\right]}^{T}$, and Num are the number of UWB anchors in the UWB positioning system. v(k) is the measurement noise and $v(k)∼N(0,r_{k})$. $\Delta d_{k,Num}(Num=1,2,\cdots )$ is the measurement information representing the difference between the UWB and IMU range values at time k.

(19) $$\Delta d_{k,Num}=|d_{i}^{imu}-d_{i}^{uwb}|$$

$d_{i}^{imu}$ and $d_{i}^{uwb}$ indicate the range values obtained by IMU and UWB, respectively.

(20) $$\left\{ \begin{array}{l} |\Delta d_{k,Num}|\geq Thr\;,\;\mathrm{NLOS}\\ |\Delta d_{k,Num}|<Thr,\;\mathrm{LOS} \end{array}\right.$$

Based on the results of the above formula, the weight matrix $\varepsilon_{k}$ of the observation residuals is obtained:

(21) $$\varepsilon_{k}=\{\begin{array}{c} e_{Num}=1,|\Delta d_{k,Num}|<Thr\\ e_{Num}=\varepsilon_{Num}\cdot \frac{\Delta d_{k,Num}}{Thr},|\Delta d_{k,Num}|\geq Thr \end{array}$$

In Equation (21), the weight matrix $\varepsilon_{k}$ is the unit matrix when the judgement is for a line-of-sight environment, and the value of $e_{Num}$ needs to be adjusted when the judgement is for a non-line-of-sight environment. Systematic prediction error update is as follows

(22) $$P_{k}=(I-\varepsilon_{k}K_{k}\cdot H_{k})P_{k}^{-}$$

where I denotes the unit matrix, and finally, the position information is updated in combination with $\varepsilon_{k}$.

(23) $$x_{k}^{ekf}=x_{k}^{ekf^{-}}+\varepsilon_{k}\cdot K_{k}(Z_{k}-H_{k}(x_{k-1}^{ekf}-x_{k-2}^{ekf}))$$

### 3.3. Positioning Feedback to Correct Distance Values

The one-dimensional filtered range values still have errors that cannot be completely eliminated, particularly the effect of non-line-of-sight factors, so a positioning feedback mechanism is set up to further suppress the effect of non-line-of-sight errors on the UWB range values. The optimal estimate of the previous state is fed back to the UWB 1D-filtered current range result to obtain $\Delta D_{k-1,i}$, which dynamically adjusts the size of the UWB current range value. $\Delta D_{k-1,i}$ is calculated as shown below.

(24) $$\Delta D_{k-1,i}=|\sqrt{∥x_{k}^{ekf}-p_{i}^{uwb}∥}|$$

(25) $$\Delta Err_{k-1,i}=|D_{k-1,i}-d_{k-1,i}|$$

Determine if the size of $\Delta Err_{k-1,i}$ is greater than a threshold Thr:

(26) $$\varepsilon_{k}=\{\begin{array}{c} e_{Num}=1,|\Delta d_{k,Num}|<Thr\\ e_{Num}=\varepsilon_{Num}\cdot \frac{\Delta d_{k,Num}}{Thr},|\Delta d_{k,Num}|\geq Thr \end{array}$$

**Figure 4:**
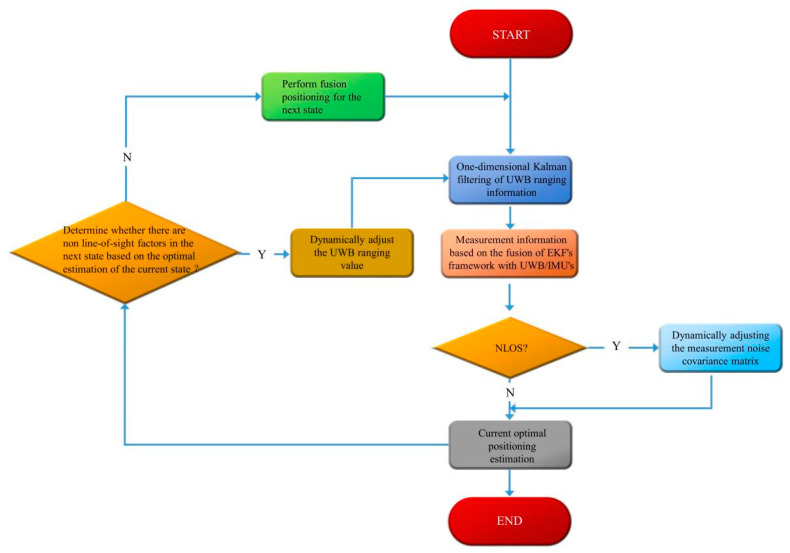
Flow chart of this method.

If it is less than the threshold Thr, then the original filtered range value is used; if it is greater than the threshold, then $D_{k-1,i}$ is used instead of $d_{k-1,i}$ to avoid the impact of non-line-of-sight errors on the positioning system. The flow of the method in this paper is shown in Figure 4.

According to the flow chart, the UWB range values are first processed by Kalman filtering, and then data fusion is performed with IMU measurement data based on the framework of extended Kalman filtering, during which the discrimination of non-line-of-sight factors is carried out, and the measurement equations in the EKF are dynamically adjusted according to the discrimination results to obtain the current state optimal positioning estimate. Due to the positioning continuity, the optimal estimate of the current state is fed back to the Kalman-filtered range values of the next state, and again the non-line-of-sight factors are discriminated, and the dual non-line-of-sight discrimination tries to suppress the influence of non-line-of-sight factors on the positioning system to improve the accuracy of the positioning system.

## 4. UWB Ranging Pre-Experiment and Analysis

In practical applications, the distance information between the multiple UWB anchors and the UWB tag needs to be obtained in a UWB positioning system in order to find the positioning coordinates through the solving algorithm. Moreover, due to the influence of external factors such as the environment, the distance measurement value of the UWB positioning sensor may have some errors, and therefore the experiment in this section collects the distance information when four UWB anchors are working simultaneously.

**Figure 5:**
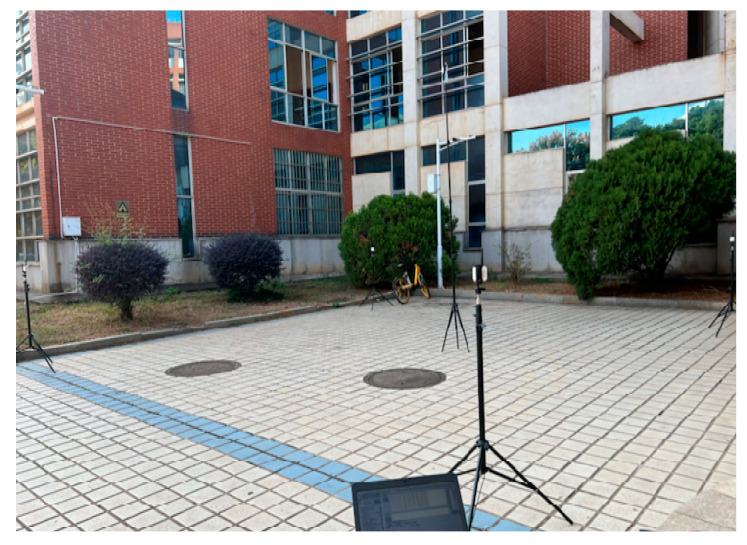
UWB ranging pre-experimental site.

The distance measurement pre-experiment site and distance measurement schematic diagram are shown in Figure 5. When the UWB module remains powered on, the PC can receive real-time measurement information from the UWB. In the experiment, the two-dimensional coordinates of the target node are fixed, and the ranging values of the target node at different heights are collected. The heights of the target node are divided into nine groups, namely 0.950 m, 1.200 m, 1.450 m, 1.700 m, 1.950 m, 2.200 m, 2.450 m, 2.700 m and 2.950 m. Each group collects 300 ranging values. The measurement data of the laser rangefinder with a measurement accuracy of 0.001 m is used as the true value of the measurement. The whole experiment is carried out in the sight distance environment.

**Figure 6:**
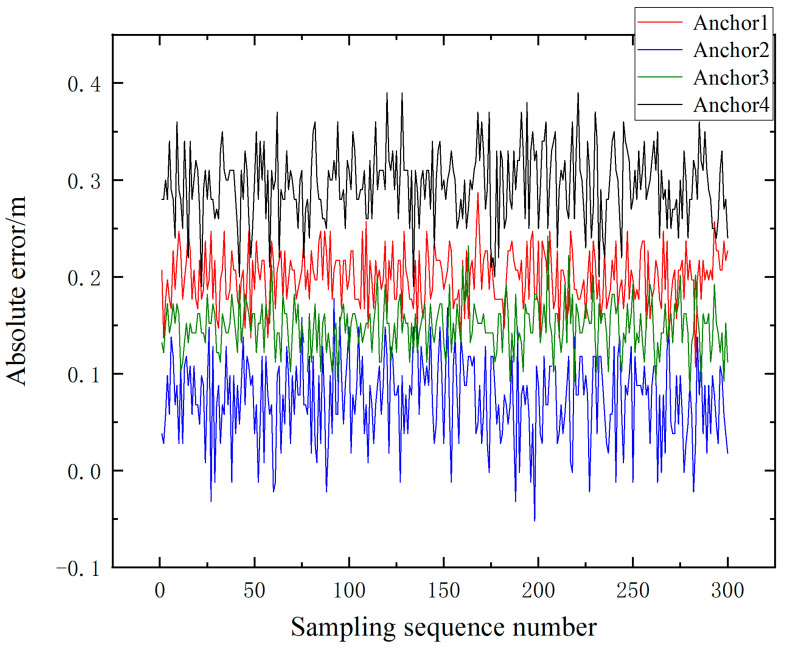
Absolute error of distance measurement of four UWB anchors.

The collected range values were compared with the true value of distance to obtain the absolute error of the range of the UWB anchors at different target node heights. The absolute error variation of the original range values of the four UWB anchors in the experimental group is shown in Figure 6, and the range accuracy of each UWB anchor is slightly different, indicating that the hardware aspects can affect the range accuracy of the UWB.

**Figure 7:**
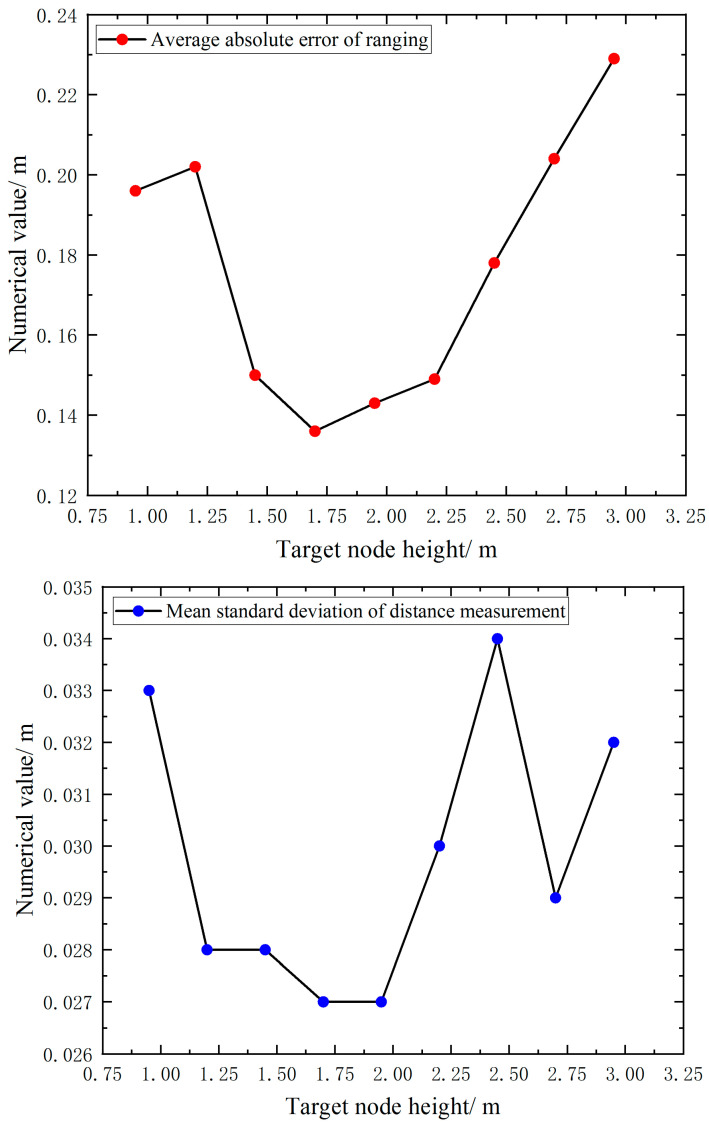
Plot of ranging error points and lines at different tag heights.

Meanwhile, the average absolute error and the standard deviation of absolute error are obtained from the absolute error statistics of ranging, as shown in Figure 7 and Table 1.

The standard deviation of the mean absolute error of the original UWB range for different target node heights fluctuates between 0.030 m. The range error of the UWB includes both systematic and random errors. Systematic errors can be dealt with by using the range error compensation model, while random errors are dealt with by using the Kalman filter due to its random nature. Through this pre-experiment, the range error pattern of the UWB during positioning is obtained, and this data will be reflected in the simulated positioning experiments, which are used to simulate the range noise of the UWB.

The pre-experiments in this section also illustrate that there is some measurement error in UWB, even in line-of-sight environments. If the UWB anchor or the UWB tag is obscured by an external object, this can prolong the time for the UWB signal to be received between the UWB anchor and the UWB tag, resulting in additional positive bias in the ranging information, so non-line-of-sight factors cannot be ignored.

## 5. Simulation Experiments and Analysis of Results

**Figure 8:**
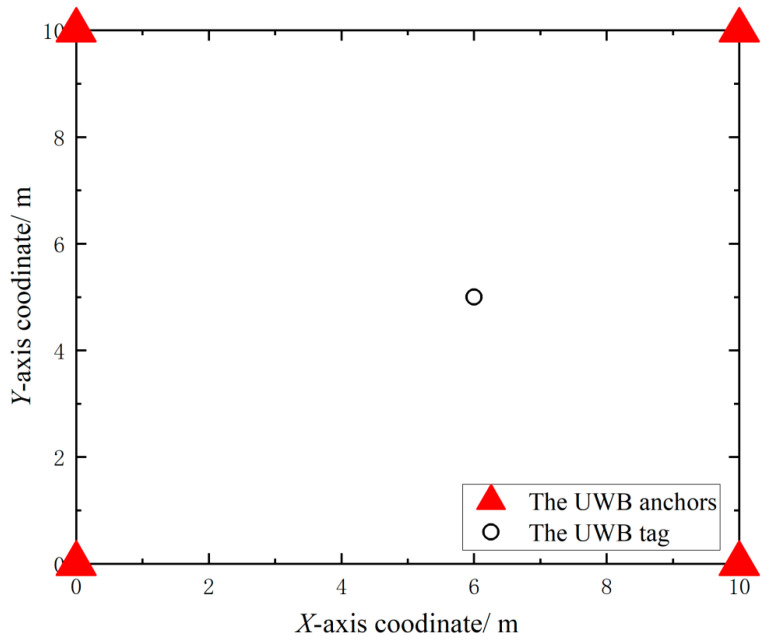
The UWB anchors deployment of the simulation positioning system.

To verify that a one-dimensional Kalman filter can effectively improve the precision of tightly coupled positioning in the line-of-sight environment by pre-processing the ranging value, this section designs a two-dimensional positioning simulation experiment on the Matlab platform and adds corresponding noise to simulate the random error in the UWB positioning environment. The random error of UWB ranging is subject to N∼(0.15,0.03). In this paper, the traditional tightly coupled positioning without one-dimensional pre-processing is assumed to be tightly coupled positioning 1, and the traditional tightly coupled positioning after ranging value pre-processing is assumed to be tightly coupled positioning 2. In this paper, the mean absolute error (MAE) is used to evaluate the estimation error of the positioning model

(27) $$MAE=\frac{1}{N}\overset{N}{\underset{i=1}{\sum }}|p_{i}-{\hat{p}}_{i}|$$

where $p_{i}$ denotes the true value of positioning and ${\hat{p}}_{i}$ denotes the estimated value of positioning. Four UWB anchors were deployed in the simulated positioning experiment with coordinates of (0, 0), (10, 0), (0, 0) and (10, 0), respectively, and the UWB anchors of the simulated positioning experiment were deployed, as shown in Figure 8.

**Figure 9:**
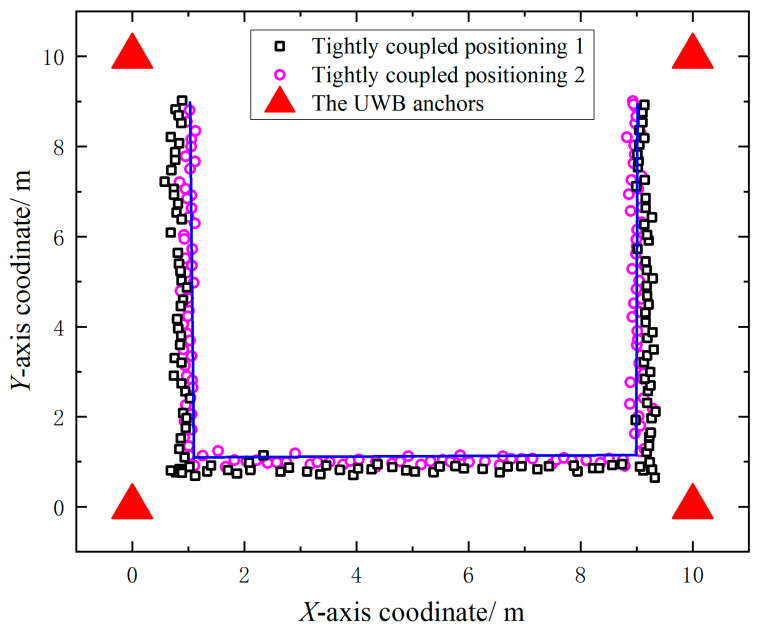
Comparison between the positioning results of two positioning methods and the real track.

In the simulation experiments, the target nodes were estimated by tightly coupled positioning 1 and tightly coupled positioning 2, respectively, according to the pre-defined trajectories with random ranging error interference. The comparison of the solution results of the two localization methods with the real trajectory is shown in Figure 9. Due to the lack of 1D data pre-processing, the localization results of tightly coupled positioning 1 are not as accurate as those of tightly coupled positioning 2, which is also closer to the real trajectory.

As shown in Table 2, the average positioning error of tightly coupled positioning 1 in the simulated positioning experiments was 0.093 m, while the positioning error of tightly coupled positioning 2 was 0.075 m. In terms of range value pre-processing, the positioning accuracy was improved by 19.35% after 1D range value pre-processing, as when preprocessing UWB ranging values using Kalman filtering, the difference between the current state and the previous state is used as a measurement vector, and the current ranging result is accurately estimated based on the system state, as close as possible to the true value of the distance, which can effectively solve the impact of abrupt changes in the UWB measurement process on the positioning system, verifying that 1D filter pre-processing can improve the positioning accuracy of tight coupling.

## 6. Field Experiment Results and Analysis

To verify the effectiveness of the algorithm, field experiments were carried out in this paper. The four UWB anchors were arranged in a square with a side length of 10 m and a fixed height of 1.2 m. Communication between the PC and the UWB tag was performed via a Bluetooth serial port, and the raw UWB and IMU measurement data were acquired in real time using STC-ISP software.

The experimental environment of this paper was the weak differential signal field of the eighth teaching building of Hunan Agricultural University, in which a two-dimensional positioning experimental platform was built and a crawler chassis cart was used as the target positioning carrier. In the experiment, the crawler chassis cart was controlled by remote sensing to travel at a constant speed and along a 6 m × 6 m square, and the data measured by the laser rangefinder were used as the true value of the distance measurement. In this case, the speed of the tracked chassis was controlled at 0.45 m per second.

This test determines the true track by controlling the track and speed of the crawler chassis cart and jointly using the Laser rangefinder. We set the rectangular motion track and controlled the crawler chassis cart to travel in a straight line. We used the Laser rangefinder to calibrate at the four vertices of the rectangle to determine the true travel track of the crawler chassis cart.

**Figure 10:**
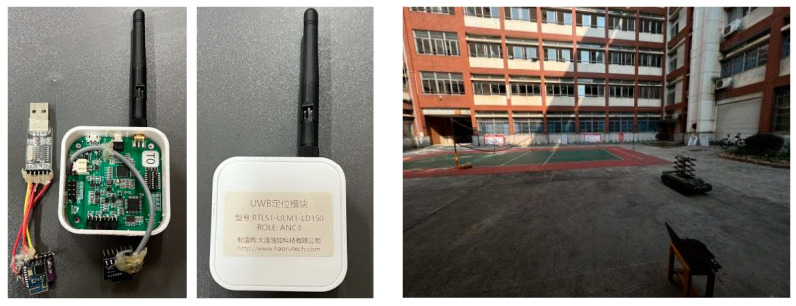
The UWB tag and Bluetooth serial port module; the UWB anchors module; and experimental site.

When the trolley moved between the UWB anchors with coordinates (0 m, 0 m) and (0 m, 10 m), an obstruction was placed in front of both as a non-visual interference factor. The positioning system module, as well as the experimental site, are shown in Figure 10, and the measurement data acquisition frequencies of both UWB and IMU sensors are synchronized with values of 9–10 Hz.

**Figure 11:**
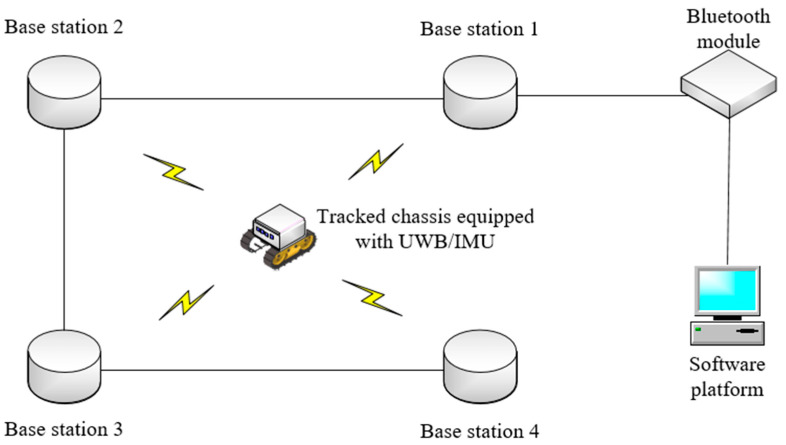
General working diagram of the UWB/IMU positioning system.

As shown in Figure 11, the overall working diagram of the UWB/IMU positioning system shows that the UWB tag is equipped with an inertial measurement element IMU to move within the UWB positioning system. The positioning information of the measured target node is obtained in real time through the controller, which is connected to the PC through Bluetooth and the UWB and IMU measurement modules.

**Figure 12:**
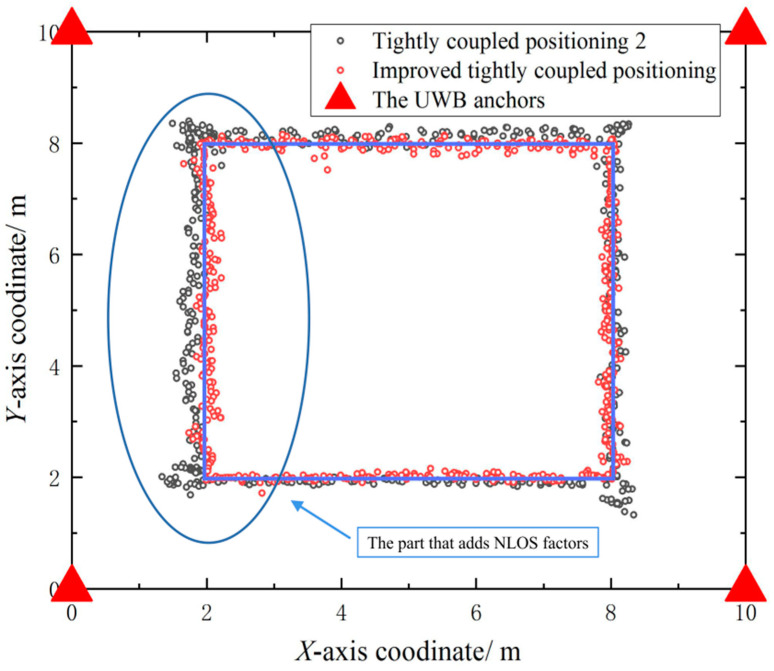
Comparison between the positioning results of different algorithms and the real trajectory.

Figure 12 shows the comparison between the positioning results of conventional tightly coupled positioning 2 and the algorithm in this paper, as well as the real trajectory, with the rectangle in the figure indicating the real trajectory of the car. It can be seen that in the line-of-sight case, both localization results are close to the true trajectory, despite the lack of a positioning feedback mechanism in tightly coupled positioning 2; that is, the current state estimation positioning does not dynamically adjust the UWB one-dimensional ranging value, so even in the case of line-of-sight, the positioning estimation results still have significant positioning errors. In a non-line-of-sight environment, due to the absence of a non-line-of-sight judgment mechanism for tightly coupled positioning 2, its estimated localization begins to deviate significantly from the true trajectory. The positioning method in this paper has dual non-line-of-sight discrimination, which can minimize the impact of non-line-of-sight factors on positioning, so it is still close to the real trajectory.

Table 3 shows the error summary of the dynamic positioning experiments. In Table 3, it can be seen that due to the non-line-of-sight error discrimination and positioning feedback mechanism, the accuracy of this method can be controlled to within 6 cm.

## 7. Conclusions

In this paper, a positioning improvement strategy is proposed for the application of tightly coupled UWB/IMU positioning to address the random errors and interference from non-line-of-sight factors in the UWB measurement process. In order to better meet the needs of practical applications, this paper first implements UWB signal ranging pre-experiments and analyses the ranging error law of UWB. Then, through the analysis of the positioning simulation results, it is determined that the pre-processed tightly coupled positioning 2 is significantly more accurate and closer to the real positioning trajectory than the traditional tightly coupled positioning 1, thus verifying that one-dimensional filtering can effectively improve the accuracy of tightly coupled positioning. For the non-line-of-sight factors, this paper improves on the tightly coupled positioning 2 by introducing a feedback mechanism for determining the non-line-of-sight factors and optimal positioning estimation, and verifying it through field experiments. The experimental results show that the accuracy of the improved strategy is significantly improved compared to the tightly coupled positioning 2. The simulation and field experiments show that the improved positioning strategy can effectively improve the positioning accuracy of tightly coupled UWB/IMU positioning.

This study focuses on UWB/IMU tight coupling in two-dimensional positioning applications. In subsequent research, it will also consider:

(1) Studying the application of UWB/IMU tightly coupled positioning in three-dimensional positioning, which will involve positioning algorithms for three-dimensional coordinates and solutions for non-line-of-sight problems that cater to three-dimensional positioning.
(2) UWB/IMU tightly coupled positioning uses only two types of positioning sensors, reducing the economic cost of positioning. In order to expand the application range of the combined positioning system and increase the stability of the positioning system, other positioning sensors such as laser radar, GNSS and machine vision can be considered for future research.
(3) In this paper, UWB and IMU measurement data are fused under the framework of EKF to obtain the location of the target node. Future research will consider improving the fusion algorithm to improve the robustness of its location algorithm.

## Figures and Tables

**Table 1:** Distance measurement error data at different UWB tag heights.

Data Type/m	Target Node Height/m								
	0.950	1.200	1.450	1.700	1.950	2.200	2.450	2.700	2.950
Average absolute error of ranging	0.196	0.202	0.150	0.136	0.143	0.149	0.178	0.204	0.229
standard deviation	0.033	0.028	0.028	0.027	0.027	0.030	0.034	0.029	0.032

**Table 2:** Error comparison table of simulation positioning.

Positioning Method	*X*-Axis Positioning Error	*Y*-Axis Positioning Error	Mean Absolute Error
Tightly coupled positioning 1	0.087	0.098	0.093
Tightly coupled positioning 2	0.074	0.078	0.075

**Table 3:** Error comparison between traditional tightly coupled positioning and this method.

Positioning Methods	*X*-Axis Positioning Error	*Y*-Axis Positioning Error	Mean Absolute Error
Tightly coupled positioning 2	0.097	0.085	0.091
Improved tightly coupled positioning	0.041	0.058	0.049

## Data Availability

Not applicable.
